# PET/CT-Based Salvage Radiotherapy for Recurrent Prostate Cancer After Radical Prostatectomy: Impact on Treatment Management and Future Directions

**DOI:** 10.3389/fonc.2021.742093

**Published:** 2021-08-31

**Authors:** Jennifer le Guevelou, Vérane Achard, Ismini Mainta, Habib Zaidi, Valentina Garibotto, Igor Latorzeff, Paul Sargos, Cynthia Ménard, Thomas Zilli

**Affiliations:** ^1^Division of Radiation Oncology, Geneva University Hospital, Geneva, Switzerland; ^2^Division of Radiation Oncology, Centre François Baclesse, Caen, France; ^3^ Faculty of Medicine, Geneva University, Geneva, Switzerland; ^4^Division of Nuclear Medicine and Molecular Imaging, Diagnostic Department, Geneva University Hospital, Geneva, Switzerland; ^5^Geneva Neuroscience Center, Geneva University, Geneva, Switzerland; ^6^Department of Nuclear Medicine and Molecular Imaging, University of Groningen, University Medical Center Groningen, Groningen, Netherlands; ^7^Department of Nuclear Medicine, University of Southern Denmark, Odense, Denmark; ^8^Department of Radiation Oncology, Groupe Oncorad-Garonne, Clinique Pasteur, Toulouse, France; ^9^Department of Radiation Oncology, Institut Bergonié, Bordeaux, France; ^10^Department of Radiation Oncology, Centre Hospitalier de l’Université de Montréal (CHUM), Montréal, QC, Canada

**Keywords:** prostate cancer, relapse, radiotherapy, PET/CT, PSMA, choline, fluciclovine, oligometastatic

## Abstract

Biochemical recurrence is a clinical situation experienced by 20 to 40% of prostate cancer patients treated with radical prostatectomy (RP). Prostate bed (PB) radiation therapy (RT) remains the mainstay salvage treatment, although it remains non-curative for up to 30% of patients developing further recurrence. Positron emission tomography with computed tomography (PET/CT) using prostate cancer-targeting radiotracers has emerged in the last decade as a new-generation imaging technique characterized by a better restaging accuracy compared to conventional imaging. By adapting targeting of recurrence sites and modulating treatment management, implementation in clinical practice of restaging PET/CT is challenging the established therapeutic standards born from randomized controlled trials. This article reviews the potential impact of restaging PET/CT on changes in the management of recurrent prostate cancer after RP. Based on PET/CT findings, it addresses potential adaptation of RT target volumes and doses, as well as use of androgen-deprivation therapy (ADT). However, the impact of such management changes on the oncological outcomes of PET/CT-based salvage RT strategies is as yet unknown.

## Introduction

Between 20 and 40% of patients treated with radical prostatectomy (RP) will develop biochemical recurrence (BCR) (1–3), defined as a confirmed rising prostate specific antigen (PSA) during the postoperative follow-up (4). The risk is greater among patients with high-risk features, such as extraprostatic extension, seminal vesicles invasion, or positive surgical margins (5). Four randomized trials have shown a twofold reduction in BCR with adjuvant radiotherapy (RT) compared with observation for patients with high-risk features (6–9), resulting in a potential improvement in both metastasis-free survival and overall survival (10). Still, adjuvant RT has been withdrawn in favor of early salvage radiotherapy (SRT), associated with the same oncological benefit for the majority of relapsing patients without high-risk features (11–14), when performed at low PSA values (15). Furthermore, this shift in practice avoids the use of immediate adjuvant RT, and the associated toxicity, in approximately 40% of patients (12).

The role of restaging imaging is not clearly defined in the salvage setting, and current guidelines recommend irradiation of the prostate bed (PB) with or without the whole pelvis using standardized anatomic-based contouring atlases (16–18). Based on current evidence, SRT remains, however, non-curative for some patients, raising concerns about the potential role of restaging at BCR and the appropriateness of irradiation volumes and/or radiation doses used in this setting.

In the last decade, positron emission tomography with computed tomography (PET/CT) using new radiotracers has emerged in clinical practice as a new imaging modality, proving both higher sensitivity and specificity than conventional imaging in detecting recurrence after RP (19, 20). By providing more accurate staging, PET can potentially lead to significant adjustments in treatment management. Together with advances in RT techniques, PET imaging may therefore offer novel perspectives for treatment optimization, such as metastasis-directed therapy (MDT), thereby challenging the established therapeutic standards.

This narrative review aims to assess the influence of PET/CT on treatment changes for salvage postoperative radiation treatment in prostate cancer patients with BCR after RP. Potential modification of target volumes, RT doses, and use of androgen-deprivation therapy (ADT) for SRT treatments based on PET/CT findings are considered and discussed.

## Modern Imaging Modalities in Prostate Cancer: PET/CT

**Figure 1:**
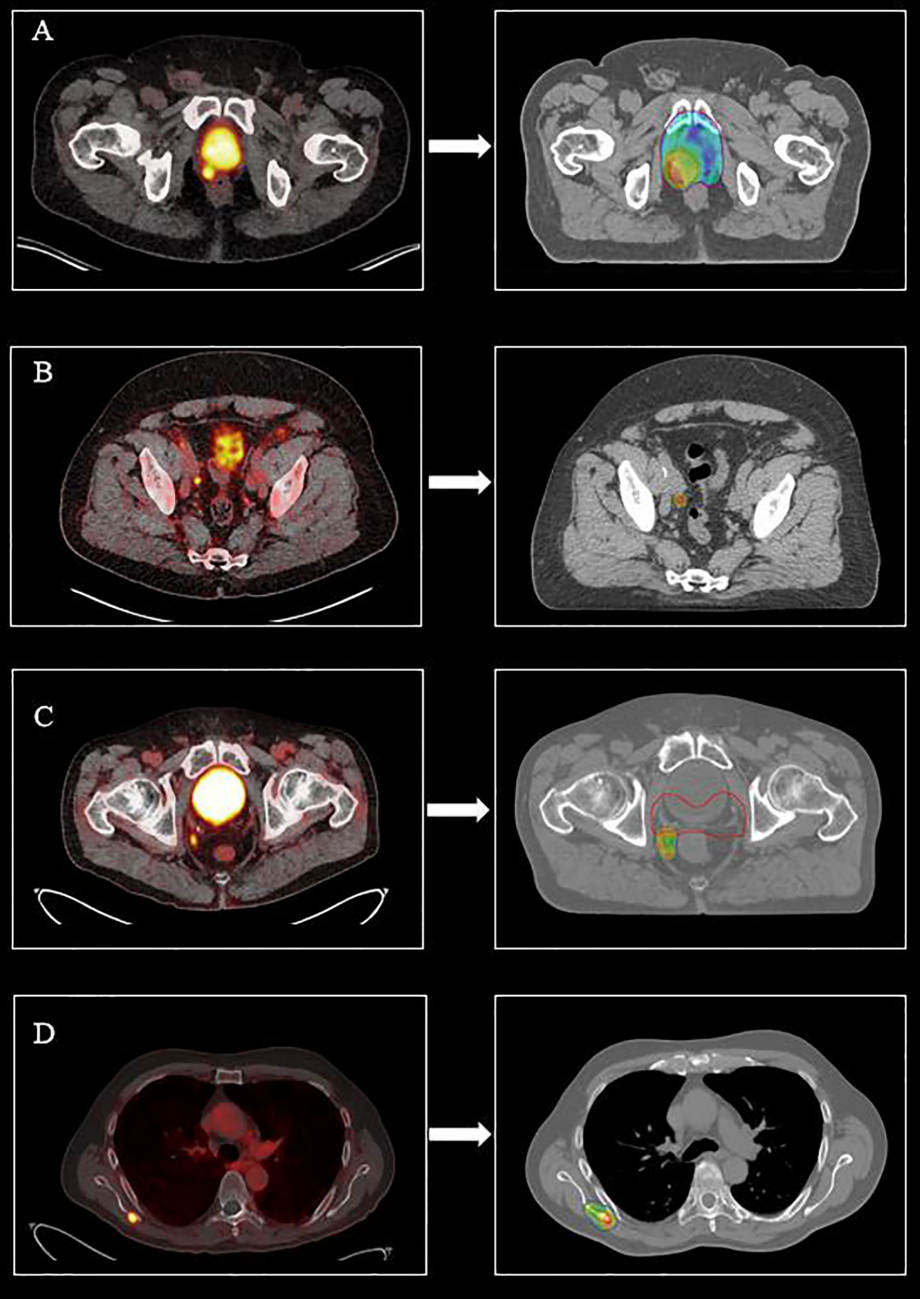
^68^Ga-PSMA PET/CT restaging findings and the corresponding salvage radiotherapy treatments (color wash isodose line 95%) in patients with biochemical recurrent prostate cancer after radical prostatectomy. **(A)**^68^Ga-PSMA PET/CT revealing a prostate bed recurrence located close to the bladder neck (left). Prostate bed radiotherapy (64 Gy/32 fx) planned with a simultaneous integrated boost (70.4 Gy/32 fx) (right) to the PET/CT positive lesion. **(B)**^68^Ga PSMA PET/CT revealing a millimetric solitary right external iliac node (left). Salvage stereotactic body radiation therapy planned on the PSMA avid node (30 Gy/3 fx) (right). **(C)**^68^Ga PSMA PET/CT revealing a perirectal oligorecurrent nodal relapse after radical prostatectomy and salvage prostate bed radiotherapy (left). Stereotactic body radiation therapy planned on the PSMA avid node (35 Gy/5 fx). Prostate bed PTV is shown in red (right). **(D)**^68^Ga PSMA PET/CT revealing an oligometastatic bone metastasis located at the right scapula (left). Stereotactic body radiation therapy planned to the PET/CT positive bone lesion (30 Gy/3 fx).

The benefit to performing local treatment such as RT is critically dependent upon imaging methods and its accuracy to assess disease at local, nodal, and metastatic level (**Figure 1**). Traditionally, computed tomography (CT) and bone scintigraphy have been used for both staging and follow-up of patients with prostate cancer, yet they often lead to understaging. Indeed, CT was shown to have a 32% sensitivity only in detecting nodal metastases in a meta-analysis led by Hövels et al. (19), with both sensitivity and specificity dropping precipitously at low PSA levels, when indication for SRT is usually undertaken. Bone scintigraphy remains the standard for the detection of bone lesions, but pooled results from a meta-analysis revealed a sensitivity and specificity of 59 and 75%, respectively (20).

Radiolabeled choline is one of the most extensively studied tracers in the restaging of prostate cancer in BCR (21). It is a substrate for choline kinase, upregulated in rapidly duplicating cells to meet the increased demands for membrane phospholipid synthesis, a biomarker associated with cell proliferation (22). ^18^Fcholine PET/CT was found to have a diagnostic accuracy of 84%, with a sensitivity of 79% and a specificity of 97% in bone evaluation (23), while its sensitivity ranges from 33 to 100% for nodal disease assessment, with a specificity of 97% (24). Most of its limitations come in the restaging performance of patients with a PSA level <2 ng/ml and with a doubling time >6 months (25). Still, European Association of Urology (EAU) guidelines recommend to perform choline PET/CT at BCR if PSA value is >1 ng/ml (4).

^18^F-Fluciclovine PET/CT is also indicated at BCR, after primary treatment with curative intent (26, 27). It has the ability to detect amino acid transport, which is upregulated in numerous types of cancer cells (28). Fluciclovine PET/CT was found to be both more sensitive (45 *vs* 21%) and more specific (29 *vs* 14% at PSA values <1 ng/ml) than choline PET/CT (29), and thus received approval by the Food and Drug Administration (FDA) in the recurrent setting. In the phase III FALCON trial, the detection rate of Fluciclovine was 56% at a median PSA level at restaging of 0.79 ng/ml (30).

**Figure 2:**
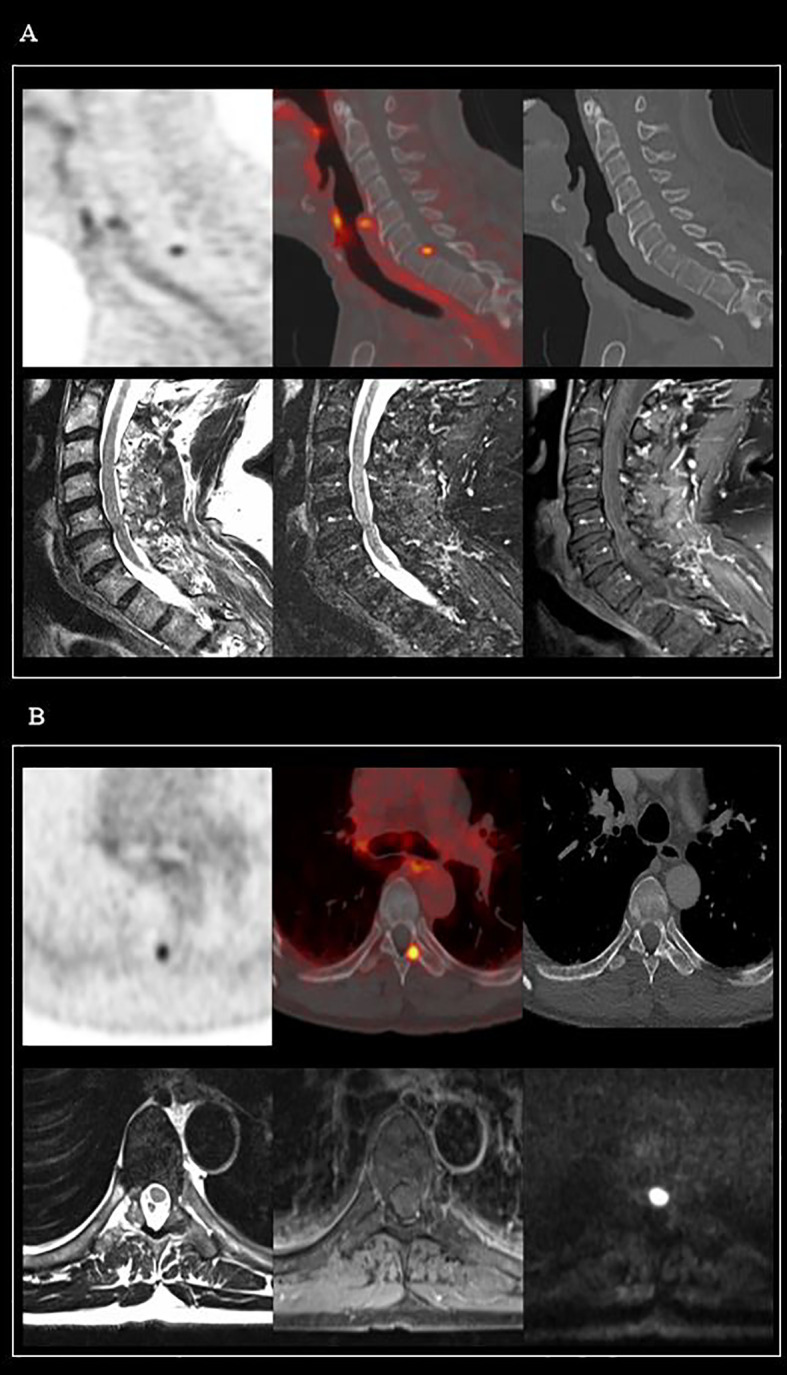
Two cases of PSMA-avid bone uptakes, with no evidence of metastatic lesions on MRI imaging. **(A)** ^68^Ga-PSMA PET/CT: Millimetric and PSMA-avid bone lesion on the posterior part of the vertebral body of C7. MRI (from left to right): T2 TSE, T2, and T1 TSE FS Gadolinium MRI sequences, all in favor of a benign bone lesion. **(B)** ^68^Ga-PSMA PET/CT: Avid bone lesion at the base of the left transverse process of the D7 vertebra. MRI (from left to right): T2 TSE, T1 TSE FS Gadolinium, and b800 diffusion MRI sequences, all in favor of a benign bone lesion.

PET/CT using Prostate Specific Membrane Antigen (PSMA) radiotracer, either radiolabeled with ^68^Ga or ^18^F, detects cellular expression of PSMA and is being increasingly used in the staging of prostate cancer patients (31, 32). In a population of high-risk localized prostate cancer, PSMA PET/CT was shown to have a 27% greater accuracy than conventional imaging (92 *vs* 65%, p<0.0001), with a sensitivity of 85% and a 98% specificity (33). In the recurrent setting, PSMA PET/CT showed excellent detection rates even at very low PSA values (42% for PSA levels ≤0.2 ng/ml) (34). Still, cautious evaluation is required in case of solitary PSMA-avid lesions, especially on the bones, as they may be false-positive findings (**Figure 2**) (35). Currently, EAU guidelines recommend performing PSMA PET/CT at BCR if the PSA level is >0.2 ng/ml and if the results will influence subsequent treatment decisions (4). Other jurisdictions have neither approved nor funded PSMA PET/CT given an absence of evidence demonstrating impact on improved patient outcomes.

## Local Relapse

Radiation therapy to the PB is the standard salvage therapy in men who have developed BCR after RP (36). Areas deemed at risk of local recurrence include the vesicourethral anastomosis, the retrovesical region, and the bladder neck (37). Still, despite the performance of SRT, up to 8% of the patients will develop local recurrence (38), highlighting the importance of both an adequate radiation dose and an accurate clinical target volume (CTV) definition.

To date, several guidelines have been published to standardize postoperative target volume: the Radiation Therapy Oncology Group (RTOG) (39), the European Organisation for Research and Treatment of Cancer (EORTC) (17), the Faculty of Radiation Oncology Genito-Urinary Group (FROGG) (40), the Genito Urinary Radiation Oncologists of Canada (GUROC) (41), and the Francophone Group of Urological Radiation Therapy (GFRU) (16). These standardized volumes do not, however, cover all potential sites of recurrence. In a study assessing the patterns of local relapse in patients with BCR after RP, the anastomosis was the most common site of recurrence (52.8%) identified by ^18^F-Choline PET/CT, followed by the retrovesical region (31.7%) and the bladder neck (7%) (42). Eighty-four percent and 83% of local relapses were entirely included in CTV, defined according to RTOG and FROGG guidelines, respectively. This rate was significantly lower using the EORTC guidelines (68%, p=0.006), due to a lack of coverage of the bladder neck and the retrovesical region. Still, 60% of relapses occurring in the posterior region of the anastomosis were not covered by any of the CTVs. Extending the target volumes in a standardized manner would necessarily result in an increased dose to organs at risk (OAR), which may ultimately increase the risk of late toxicity, particularly in the urinary tract. On the other hand, personalization of target volumes and radiation doses by implementing restaging PET/CT can potentially improve the therapeutic ratio of recurrent patients who are candidates for SRT.

Management of local macroscopic recurrence after RP is characterized by a high variability of treatment paradigms (43). Use of focal boost with or without whole pelvis irradiation (44), addition of concomitant short-term or long-term ADT (45, 46), or delivery of a focal SBRT to the macroscopic relapse (47) have been hypothesized as possible alternatives to SRT to the PB only.

A focal boost on the PET-positive local recurrence represents one the mostly studied potential contributions of functional imaging. In a study including 60 patients, D’Angelillo et al. reported a focal boost of up to 80 Gy to a biological target volume (BTV) defined by ^18^F-Choline PET/CT. The 3-year biochemical progression-free survival rate was 72.5%, with only three patients experiencing grade 3 acute gastrointestinal toxicity, and no grade 3 late toxicity (48). Still, detection of local recurrence remains challenging as ^18^F-Choline PET/CT suffers from a low spatial resolution with inconsistent sensitivity in this setting, ranging from 64% to 100% (25). In addition, the low sensitivity of choline PET at PSA values <2 ng/ml and in case of doubling time >6 months makes this diagnostic modality poorly suitable for patients eligible for early SRT. At low PSA values, PSMA PET/CT appears to be the best diagnostic option, with detection rates of about 50% at PSA levels of less than 0.5 ng/ml (49, 50). Calais et al. reported the diagnostic performance of PSMA PET/CT and Fluciclovine PET/CT in a population of patients with BCR after RP (51). Detection rates were significantly lower with ^18^F-Fluciclovine PET/CT than with PSMA PET/CT (26 *vs* 56%, p=0.0026). However, on a local level, the assessment by PSMA PET, especially with ^68^Ga, may be limited due to urinary excretion of the tracer. This was emphasized in a study conducted by Pernthaler et al. (52) in a population of patients with BCR, where a higher rate of prostatic recurrence was found with Fluciclovine PET than PSMA PET (37.9 *vs* 27.6%, p=0.03). In the EMPIRE-1 trial (53), prostate cancer patients with BCR were randomly assigned in two arms: the first received SRT based on conventional imaging, the second underwent Fluciclovine PET/CT, and treatment was planned according to those findings. In case of pelvic nodal uptake, patients received PB and pelvic RT, with nodal boost up to 54–56 Gy. In case of PB-only uptake, patients received PB RT, with a boost up to 76 Gy on the local recurrence. When no uptake was found, patients were treated on the PB only. The 3-year failure-free survival was superior in patients treated with Fluciclovine PET/CT-guided SRT compared to patients treated using conventional imaging only (75.5 *vs* 63%). This difference widened at the 4-year evaluation (75.5 *vs* 51.2%, p=0.001) (54), which can be attributable to both a stage-migration phenomenon and a reduction of the in-field relapses related to the SRT dose escalation. Regardless of some inherent limitations [low proportion of patients receiving whole pelvis RT (WPRT), 25% of patients with a PSA level >1 ng/ml at salvage, lack of intent-to-treat analysis], this study can be considered hypothesis generating with respect to a possible improvement in outcomes with PET-guided SRT.

In an attempt to further reduce toxicity and improve outcome, focal treatments directed to the local PB relapse using modern SBRT techniques have been proposed as the last frontier of SRT (47), with promising preliminary results requiring, however, further prospective validation.

Isolated macroscopic local recurrence after RP remains a rare situation, representing 12% of BCR cases in a study by Calais et al. using restaging PSMA PET/CT on a population with a PSA value <1 ng/ml (55). Whether or not to treat pelvic lymph nodes in this setting remains an open question. In an analysis of recurrence patterns after PB RT, Douglas et al. reported up to a 39% of isolated pelvic nodal failure after SRT to the PB (56). To date, in this population of relapsing patients after RP, the results of the NRG Oncology/RTOG 0534 SPPORT trial demonstrated the superiority of WPRT + PB RT over PB RT alone, both combined with a short course of ADT (44). Exploratory subgroup analyses of this trial suggested that the benefit for nodal irradiation was more pronounced for men with a PSA >0.34 ng/ml at the time of salvage treatment. However, restaging modalities used in this study were not based on modern imaging. Irradiating or not the whole pelvis in node-negative PSMA patients remains therefore an open question, requiring prospective evaluation. Noteworthy, even with PSMA PET/CT, the detection of lymph node metastases is moderate (33–91%), due to the inherent limitations in spatial resolution to detect small (<3 mm) nodal metastases (57). Besides, men at high-risk of micro-metastatic nodal involvement are probably the most likely to benefit from WPRT (58).

## Nodal Relapse

Lymph nodes are commonly identified as a site of failure in prostate cancer, particularly in the post-RP setting, followed by distant bone metastases (59). Although nodal relapses after RP follow common patterns of disease spread in the majority of the cases, a relevant percentage of patients exhibit nevertheless an aberrant nodal spread. In a study of Meijer et al. using magnetic resonance lymphography (60), 79% of the patients presented an aberrant lymph node spread, most of them being located in the perirectal region and in the para-aortic region. Using PSMA PET/CT in a population of patients with PSA <1 ng/ml after RP, Calais et al. also supported the finding that perirectal lymph nodes are the most common site of nodal recurrence outside the pelvic CTV (55). Implementing PET/CT studies data on patterns of nodal relapse, recent guidelines as the NRG Oncology Updated International Consensus atlas recommend cranial extension of CTV volumes to include the common iliac nodes (18). Inclusion of peri-rectal nodes in the CTV volume remains a source of discussion, especially for T4 tumors (61).

PET/CT imaging can be used to provide guidance for the realization of a boost, in case of intrapelvic nodal recurrence. Fodor et al. reported the 3-year toxicity and outcomes of a choline PET/CT-guided RT in patients with a nodal relapse. Pelvic and/or lombo-aortic irradiation was performed at 51.8 Gy/28 fractions, with simultaneous integrated boost (SIB) technique to a median dose of 65.5 Gy on the pathological uptake sites. Ninety-one percent of the patients had a PSA reduction 3 months after RT, with a 3-year clinical relapse free survival of 61.8% (62). The single-arm phase II Oligopelvis-GETUG P07 trial also explored the role of concomitant salvage pelvic irradiation with moderate hypofractionation (54 Gy/30 fractions to the pelvis, 66 Gy/30 fractions the lymph nodes) in combination with 6 months of ADT. A persistent complete biochemical response was found in 73.1 and 45.9% of the patients at 2 and 3 years, respectively, with a 2-year progression-free survival (PFS) of 77.6% (63, 64).

MDT strategies using stereotactic body radiotherapy (SBRT) have been widely used as an alternative to elective nodal irradiation. Many teams reported on outcomes after MDT alone, enrolling heterogeneous populations with both nodal and bone metachronous metastases, and both ADT and non-ADT treated patients. Local control and ADT-free survival were the most common endpoints. The STOMP trial (65) randomized patients with one to three metastases (55% of the patients with nodal disease, 45% with bone metastases) detected by Choline PET/CT studies to MDT (SBRT or salvage lymph node dissection, sLND) or observation. Median ADT-free survival was in favor of performing MDT (21 months *vs* 13 months), with a greater benefit among patients with PSA doubling time <3 months. Similarly, the ORIOLE study confirmed the benefit of MDT in terms of biochemical control in a population of recurrent patients diagnosed with oligometastatic disease by PSMA PET/CT (66). The proportion of men with disease progression at 6 months was 19% among patients treated with MDT compared to 61% in the observation arm (p=0.005). Of note, total consolidation of PSMA -avid lesions decreased significantly the risk of new lesions at 6 months (16 *vs* 63%). Although results of MDT studies are encouraging, whether or not to perform WPRT in combination with MDT remains an unresolved issue with large variability in the treatment volumes proposed in patients with oligorecurrent nodal disease (67). Initial series with choline PET/CT seemed to discourage the planning process of MDT on only positive spots (68). Even when PSMA PET/CT is used, surgical series have showed that bilateral and extended treatment of nodal disease is more likely to provide complete biochemical response than targeted node dissection. In a study by Siriwardana et al. (69), 90% of patients achieved a biological complete response after bilateral sLND compared with 33.3 and 21.4% in those undergoing unilateral and targeted node dissection, respectively. Also, Ploussard et al. reported after sLND heterogeneous results in terms of biochemical progression-free survival, ranging from 23 to 64% at 2 years (70). In analogy, subsequent relapses after SBRT for oligometastatic nodal recurrences are again nodal and oligometastatic (71). Despite better results compared with choline PET/CT, the sensitivity of PSMA PET/CT seems insufficient to warrant the performance of focal nodal MDT, in order to obtain biological complete response. Further insights into the benefit and toxicity of elective nodal irradiation will be provided by the results of the PEACE V - STORM prospective randomized phase II trial, assessing the potential of combined WPRT and MDT as compared to MDT alone on metastasis-free survival of patients with nodal oligorecurrent prostate cancer (72, 73).

## Extrapelvic Oligometastatic Relapse

Implementation of PET restaging in the therapeutic workflow of prostate cancer patients relapsing after RP can lead to a modified TNM staging in up to 45.2% of the patients in comparison with conventional imaging modalities (74). In most of the cases, PSMA PET/CT upstages a subset of patients to an M1 status who otherwise would be staged M0 by conventional imaging (75). By modifying the treatment management in about half of the situations (27, 30, 32, 34), and individualizing RT volumes (**Table 1**), PET/CT imaging may play a role for a better selection of patients eligible for local salvage therapies. Still, caution is needed when modifying treatment strategy (particularly from a curative to a palliative intent), as PET/CT restaging may be questioned on its strong evidence of improved therapeutic outcomes (76). Particularly in situations where the discovery of a metastatic lesion shifts the treatment towards a palliative intent, a pathological confirmation may be warranted before making any therapeutic decision.

**Table 1:** Prospective trials evaluating the management change rate after restaging PET/CT in prostate cancer patients with biochemical recurrence after radical prostatectomy.

Trial	N	Study design	Primary endpoint	Results
Andriole et al. (27) LOCATE - NCT02680041	114	Prospective trial: ^18^F-Fluciclovine	Management change post scan	- Management change: 48% (32% omission SRT; 16% change in SRT volumes)
Scarsbrook et al. (30) FALCON - NCT02578940	104 (63% RP)	Prospective trial: ^18^F-Fluciclovine	Management change post scan	- Management change: 64% - PSA response rate: 72% without PET/CT guidance *vs* 88% with PET/CT guidance
Morris et al. (32) CONDOR - NCT03739684	208	Prospective trial: ^18^F-DCFPyL (PyL) PSMA	Correct localization rate (CLR) *vs* composite standard of truth	- CLR: 84.8% to 87.0% (positive trial) - Management change: 63.9%
NCT02940262	1200, active not recruiting	Prospective trial: ^68^Ga-PSMA	Sensitivity of ^68^Ga-PSMA for detection of tumor location	- Management change: 53% (ancillary study of 161 recurrent patients) (34)

PSMA, prostate specific membrane antigen; SOC, standard of care; SRT, salvage radiotherapy; RP, radical prostatectomy.

An increase in the frequency of patients diagnosed with oligometastatic prostate cancer is expected if PET/CT is incorporated into routine care (77, 78). Evidence grows for the treatment of these lesions with MDT, such as SBRT or surgery, in association with systemic therapies or not (79, 80). Promising results of SABR-COMET showed an improved overall survival (OS) in patients randomized to receive SBRT in addition of standard of care (SOC), compared to SOC alone (41 *vs* 28 months, p=0.09). In prostate cancer, the argument of aggressive local treatments is supported by the recent finding that indolent metastatic lesions have the potential to transform and become aggressive foci of accelerated metastases (81, 82). A recent systematic review summarized the use of SBRT for PET/CT proven oligometastatic prostate cancer (83). This study demonstrated excellent local outcomes, with no local recurrences when RT dose exceeded a biologically effective dose (BED) of >108 Gy (α/β = 3 Gy). Two-year PFS was reported in seven studies, and ranged from 30 to 64 months. For patients that did not receive concomitant ADT, median ADT-free survival ranged from 12.3 months to 39.7 months.

However, despite being able to postpone systemic therapies and probably improve survival outcomes, MDT strategies remain investigational in this setting. Trials comparing MDT with or without systemic therapies with SOC treatments (84–86) are crucially needed to confirm the benefits on both PFS and OS of this emerging therapeutic strategy.

## Biochemical Relapse and Negative PET/CT Imaging

At PSA levels defining BCR after RP, detection rates of macroscopic disease are low even with PSMA PET/CT, with a 45% detection rate at PSA levels ranging from 0.2 to 0.49 ng/ml (50). Considering actual evidence converging on the inverse correlation between the PSA level at SRT and long-term disease control of SRT, guidelines recommend use of early SRT to the PB at PSA level <0.5 ng/ml, even in absence of specific target (4). Noteworthy, very early SRT (PSA 0.01 to 0.2 ng/ml) was associated with a twofold decrease in biochemical failure, use of salvage ADT, and distant metastases compared to early SRT (PSA between 0.2 to 0.5 ng/ml) (87). Similarly, Fossati et al. also concluded that SRT should be given at the earliest sign of PSA rise, and even more so in case of adverse pathological findings (pT3b/pT4, Gleason score >8, positive surgical margins) (88). Also, the kinetics of PSA rise has an impact on OS, with a significant difference between patients with a PSA doubling time of less than 10 months (36). Could the addition of PET/CT to the design of these studies have affected outcomes? At the very least, it could have enabled the distinction between patients with and without macroscopic disease, resulting in a better homogeneity of the population. It is possible that the association between PSA level at SRT and outcome may be a bias related to the presence of macroscopic disease, and thus undertreatment of a certain proportion of this population. Still, it seems intuitive that providing SRT at a time when the disease is microscopic (and therefore undetectable on PET/CT) yields better outcomes in comparison with macroscopic disease. Indeed, in the study led by Emmett et al., patients who benefited the most from PB SRT were those with a negative PET/CT, with a 3-year freedom from progression evaluated at 82.5% (89, 90). The impact of PET/CT in SRT planning on long-term clinical outcomes is currently assessed by ongoing phase III trials (**Table 2**). While awaiting the results of these studies, a negative restaging PET/CT at BCR should not delay and alter the decision to perform SRT (4).

**Table 2:** Prospective randomized trials evaluating patient outcomes after restaging PET/CT in prostate cancer patients with biochemical recurrence after radical prostatectomy.

Trial	N	Study design	Primary endpoint	Results
Jani et al. (53) EMPIRE I - NCT01666808	165	Phase II/III randomized: ^18^F-Fluciclovine guided-treatment *vs* SOC	3-yr event-free survival	- 3-yr event-free survival: 63% SOC *vs* 75.5% ^18^F-Fluciclovine PET/CT - 35% treatment change with ^18^F-Fluciclovine PET/CT
EMPIRE II - NCT03762759	140, recruiting	Phase II randomized: ^18^F-Fluciclovine *vs* ^68^Ga-PSMA	Disease-free survival	- Ongoing trial
INDICATE - NCT04423211	804, recruiting	Phase III randomized, 4 arms: Baseline ^18^F-Fluciclovine	PFS	- Ongoing trial
		- No extrapelvic uptake: ○ SOC salvage therapy (LHRH agonists + SRT) ○ SOC salvage therapy (LHRH agonists + SRT) + Apalutamide		
		- Extrapelvic uptake: ○ SOC salvage therapy (LHRH agonists + SRT) + Apalutamide ○ SOC salvage therapy (LHRH agonists + SRT) + Apalutamide +MDT		
Calais et al. (91) PSMA SRT - NCT03582774	193	Phase III randomized: ^68^Ga-PSMA guided-treatment *vs* SOC	5-yr bRFS	Change in SRT plan: 28% SOC *vs* 57% PSMA PET/CT (ASCO 2020 preliminary results) primary endpoint analysis ongoing
NCT03525288	129	Phase II randomized: ^18^F-DCFPyL PSMA guided- treatment *vs* SOC	5-yr FFS	- Primary endpoint analysis ongoing
NCT04794777	450, recruiting	Phase III randomized: PSMA (either ^68^Ga or ^18^F-1007) guided-treatment *vs* SOC	PFS	- Ongoing trial
PATRON - NCT04557501 (definitive and salvage setting)	776, recruiting	Phase III randomized: ^18^F-DCFPyL PSMA guided- treatment *vs* SOC	5-yr FFS	- Ongoing trial

PSMA, prostate specific membrane antigen; SOC, standard of care; SRT, salvage radiotherapy; bRFS, biochemical relapse free-survival; RP, radical prostatectomy; MDT, Metastasis Directed Therapy; PFS, Progression Free Survival; LHRH, Luteinizing Hormone Releasing Hormone; FFS, Failure-Free Survival.

## Discussion

PET/CT is gradually being incorporated into international guidelines and is increasingly performed at various stages of the disease. ^18^F-Fluciclovine, ^68^Ga-PSMA, and ^18^F-DCFPyL PET/CT are currently approved by the Food and Drug Administration (FDA) for men with suspected prostate cancer recurrence, but worldwide approval and funding awaits evidence of improved patient outcomes.

**Figure 3:**
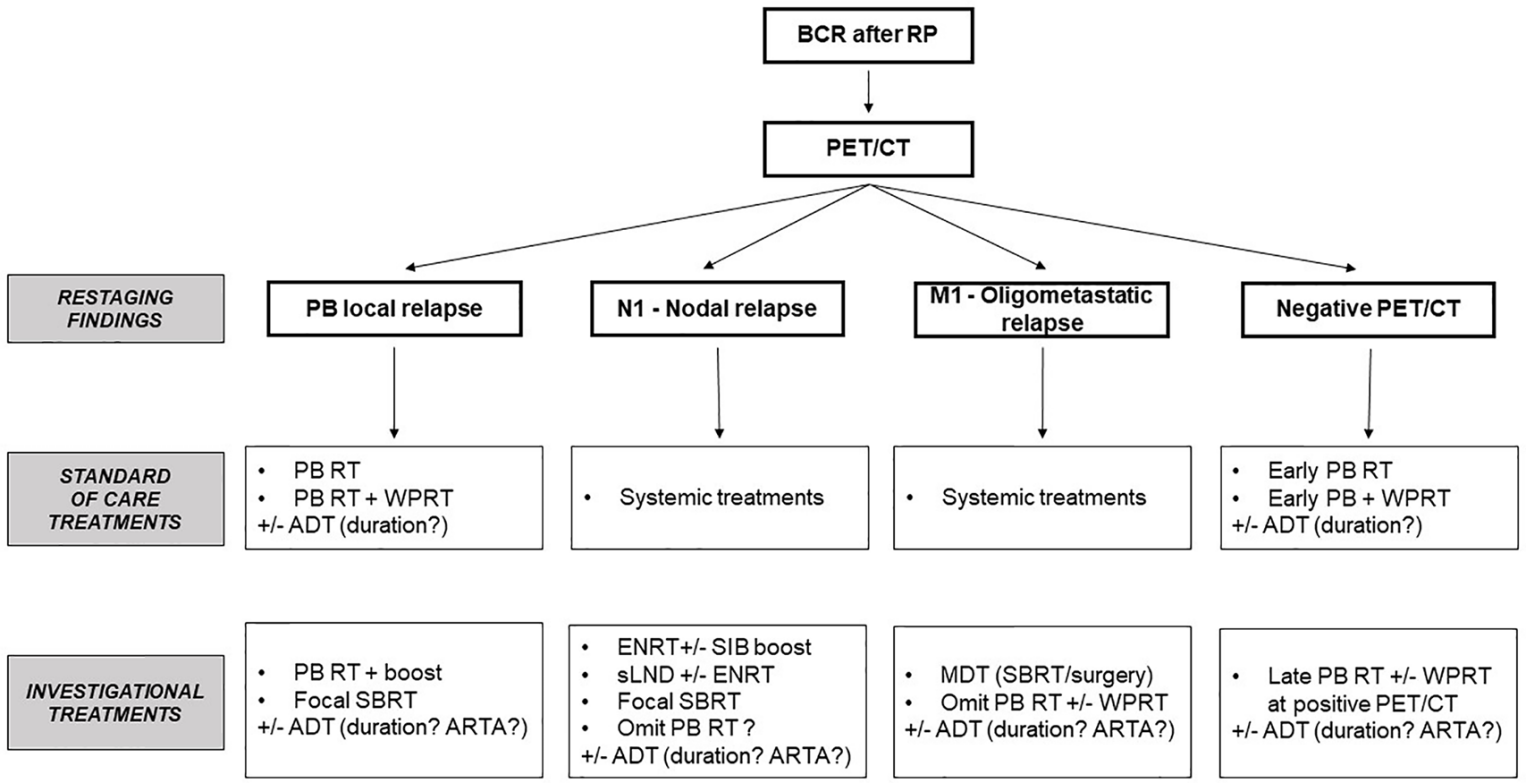
Potential therapeutic options based on PET/CT restaging findings. BCR, biochemical relapse; RP, radical prostatectomy; RT, radiotherapy; PB, prostate bed; WPRT, whole pelvic radiation therapy; SBRT, stereotactic body radiation therapy; ADT, androgen-deprivation therapy; ENRT, elective nodal radiotherapy; SIB, simultaneous integrated boost; sLND, salvage lymph node dissection; MDT, metastasis-directed therapy; ARTA, androgen receptor targeted agents.

PET/CT has proven its accuracy in restaging patients either in the local, nodal, or metastatic setting. Several studies have already proven that the implementation of PET/CT resulted in a significant management change rate in the postoperative setting, ranging from 35% (54) to 64% (30) (**Tables 1**, **2**). Still, the question whether improved staging and resultant change in management can improve clinical outcomes remains at the moment unanswered and requires confirmation in prospective trials (**Table 2**) (54, 89–91). For example, while PET/CT restaging leads us to the definition of an entirely new population of metastatic patients, their prognosis differs dramatically from the old population of metastatic patients. This effect, known as the “Will Rogers Phenomenon” (76), makes us reconsider the treatments established as a gold standard in recent years. A summary of the outstanding issues in treatment management generated by PET/CT restaging in patients with BCR after RP is provided in **Figure 3**.

Even the most accurate imaging modalities only allow us to determine the status of the disease at a given moment, without allowing us to foresee its long-term outcome. Genomic biomarkers are crucially needed in order to discriminate between an indolent or aggressive disease and provide data to guide treatment decision. Some commercially available tools have already provided new insights in identifying men with high risk of adverse outcomes (92). Cooperberg et al. demonstrated the ability of a panel of genes associated with cell cycle progression in predicting BCR after RP (93). The same panel of genes, in association with housekeeping genes, has been commercialized in the Prolaris test, which has proven its relevance in the decision of an adjuvant treatment in case of adverse pathological findings after RP (94). Some tools are also available to predict metastatic outcomes, such as Decipher tissue-based genomic classifier. Based on 22 RNA biomarkers, Decipher has proven its efficacy in predicting the 10 years’ distant metastasis (95) and prostate cancer–specific mortality (96). Molecular biomarkers thus hold the potential to select patients for appropriate treatment and thus reduce overtreatment and toxicities. One of the challenges in the future will be to identify patients with indolent disease, who will achieve satisfactory results with MDT alone, from patients with aggressive, high-risk polymetastatic disease who may benefit from the addition of systemic therapy. Recent advances in biology, such as implementation of Whole-Exome Sequencing in routine practice or understanding of microRNA pathways will probably allow us to obtain much more information on this point.

In parallel, improvements in performance of next-generation imaging including use of new prostate-specific tracers (49, 97, 98), implementation of radiomics features (99) and artificial intelligence techniques (100), and new PET imaging tools providing superior spatial and temporal resolution compared to commercially available PET scanners will undoubtedly play increasing roles in defining the presence and extent of relapsing disease and will promote the development and use of precision therapies in patients with relapsing prostate cancer.

## Conclusions

PET/CT is an emerging imaging modality with better accuracy than conventional imaging for restaging of prostate cancer patients in BCR after RP. Accurate detection of relapsing disease has led to management changes in hopes of improving the therapeutic ratio of this patient population, but to date with little evidence to support this change. Intensification of treatment strategies with delivery of focal boosts to the macroscopic relapse, expansion of target volumes to encompass areas usually not targeted by usual guidelines, addition of systemic treatments, or change in treatment intent remain open issues requiring further investigations. Ongoing trials assessing the impact of PET/CT-guided SRT will certainly help to better determine the clinical impact on long-term outcomes of integrating metabolic imaging in the restaging and therapeutic workflow of patients recurring after RP and candidates to salvage RT.

## Author Contributions

JG, PS, IL, CM, and TZ were involved in the study design and concept. JG, CM, and TZ were involved in the drafting of the manuscript. All authors contributed in the review and editing of the manuscript. All authors contributed to the article and approved the submitted version.

## Funding

This work was partially funded by a Swiss Prostate Cancer Award grant from the Movember Foundation (NCT03569241 trial), a grant from the “Fondation Privé” of Geneva University Hospital (RC06-01) and the Swiss National Science Foundation (project 320030_182366).

## Conflict of Interest

The authors declare that the research was conducted in the absence of any commercial or financial relationships that could be construed as a potential conflict of interest.

## Publisher’s Note

All claims expressed in this article are solely those of the authors and do not necessarily represent those of their affiliated organizations, or those of the publisher, the editors and the reviewers. Any product that may be evaluated in this article, or claim that may be made by its manufacturer, is not guaranteed or endorsed by the publisher.
